# Screening and identification of potential key biomarkers for glucocorticoid-induced osteonecrosis of the femoral head

**DOI:** 10.1186/s13018-022-03465-y

**Published:** 2023-01-11

**Authors:** Dan Chen, Duming Zhong, Runhong Mei, Shida Qian, Peng Wang, Kaiyun Chen, Xuefeng Yu

**Affiliations:** 1 Department of Orthopaedics, The Fourth Affiliated Hospital of Nanchang University, Nanchang, 330000 Jiangxi China; 2 Department of Drug Clinical Trial, The Fourth Affiliated Hospital of Nanchang University, Nanchang, 330000 Jiangxi China; 3 Department of Orthopaedics, Xiang Yang No.1 People’s Hospital, Xiangyang, 441100 Hubei China; 4 Department of Oral and Maxillofacial Surgery, The First Affiliated Hospital of Nanchang University, Nanchang, 330000 Jiangxi China

**Keywords:** Glucocorticoid-induced osteonecrosis of the femoral head (GIONFH), Key biomarkers, Animal model, Lipid metabolism disorder, Steroid hormones, Hypolipidemic drug

## Abstract

**Background:**

Glucocorticoid-induced osteonecrosis of the femoral head (GIONFH) is a common disease in osteoarticular surgery, with a high disability rate, which brings great physical and mental pain and economic burden to patients. Its specific pathogenesis has not been fully demonstrated, and there is a lack of recognized effective biomarkers for earlier detection and prompt treatment. This has become an urgent clinical problem for orthopedic scholars.

**Materials and methods:**

We downloaded the gene expression profile dataset GSE123568 from the Gene Expression Omnibus database, used STRING and Cytoscape to carry out module analysis and built a gene interaction network. The four core genes most related to GIONFH in this network were ultimately found out by precise analysis and animal experiment were then conducted for verification. In this verification process, thirty-six New Zealand white rabbits were randomly divided into blank control group, model group and drug group. Except for the blank control group, the animal model of GIONFH was established by lipopolysaccharide and methylprednisolone, while the drug group was given the lipid-lowering drugs for intervention as planned. The rabbits were taken for magnetic resonance imaging at different stages, and their femoral head specimens were taken for pathological examination, then the expression of target genes in the femoral head specimens of corresponding groups was detected. Validation methods included RT-PCR and pathological examination.

**Results:**

A total of 679 differential genes were selected at first, including 276 up-regulated genes and 403 down-regulated genes. Finally, four genes with the highest degree of correlation were screened. Animal experiment results showed that ASXL1 and BNIP3L were in low expression, while FCGR2A and TYROBP were highly expressed.

**Conclusion:**

Through animal experiments, it was confirmed that ASXL1, BNIP3L, FCGR2A and TYROBP screened from the comparative analysis of multiple genes in the database were closely related to GIONFH, which is important for early diagnosis of Glucocorticoid-induced osteonecrosis of the femoral head.

## Introduction

Glucocorticoids, a form of steroid hormones, are mainly used in the treatment of autoimmune diseases, such as rheumatoid arthritis, systemic lupus erythematosus and scleroderma, as well as organ transplantation and severe infection. Long-term or extensive use of glucocorticoids will bring more complications to patients, among which GIONFH is an example [1–4]. As a common type of nontraumatic osteonecrosis, GIONFH may result in the collapse of the subchondral bone structure without immediate treatment. Previous studies have shown that steroid hormones therapy can increase the risk of osteonecrosis by 4–50% [5].

In recent years, more attention has been gradually paid to GIONFH [6–8]. In the fight against Severe Acute Respiratory Syndrome (SARS) in 2003, glucocorticoids were used at high doses to save a large number of critically ill patients. Unfortunately, these steroid hormones also had side effects—the incidence of GIONFH was as high as 24–30% [9–11]. As for the novel coronavirus disease 2019 (COVID-19) epidemic, which is similar to SARS, steroid hormone is still an important medication for the treatment of infections to rescue lives, who will also be at risk of developing GIONFH in the future[12–14]. Therefore, early diagnosis and timely prevention and treatment of GIONFH have important clinical significance.

However, the pathogenesis of GIONFH is yet to be seen. The hypotheses proposed by researchers mainly include as follows-intravascular coagulation, vascular endothelial injury, increased intraosseous pressure, lipid metabolism disorder etc. [15]. Among them, the disorder of lipid metabolism is an important manifestation in GIONFH [16, 17]. It was confirmed in previous studies that steroid use may result in changes in the lipid metabolism, which increase the risk of fat embolism[18, 19]. Our team's studies also confirmed that GIONFH is closely related to the pathogenesis of lipid metabolism disorder induced by high-dose hormone in vivo[20–22]. Therefore, in order to further confirm the association between the onset of GIONFH and lipid metabolism disorder, a hypolipidemic drug group was added as comparative research to the Glucocorticoid-induced femoral head necrosis model rabbits in the animal experiment of this study.

In the current clinical research, there is a lack of methods and standards for early diagnosis of GIONFH. It is asymptomatic during the early phases. The first symptom is pain in the hip, which tends to be in proportion with lesion size, and in general precedes the beginning of femoral head collapse, which occurs on average after 8 months [23, 24]. At the moment of hip pain and dysfunction, and obvious osteonecrosis and collapse of the femoral head appear in pathological and imaging examinations, in which case surgical treatment is badly needed [25]. With the wide application of bioinformatics and the continuous development of microarray technology at the genome level, researchers have sequenced more and more disease samples with increasing fragment information, and the public functional genomics database has been greatly enriched [26, 27]. By screening and mining the massive information, we can find the mechanism of the occurrence and development of non-tumor diseases from the gene level, which helps us to screen out potential biomarkers. Then we verified the key markers screened before through animal experiments, it is expected to find new methods to facilitate the early diagnosis, detection and prevention of GIONFH.

## Materials and methods

### Gene chip data

GEO (http://www.ncbi.nlm.nih.gov/geo) is a public functional genomics database with high storage throughout gene expression data, chips, and microarrays [28]. The gene expression profile dataset GSE123568 was downloaded from the GEO database, and probes were converted to corresponding gene symbols based on annotation information on the platform [29]. The GSE123568 dataset contained tissue specimens from 30 GIONFH patients and 10 non-GIONFH patients. GEO2R (http://www.ncbi.nlm.nih.gov/geo/geo2r), an interactive web tool, was used to screen DEGs between GIONFH and non-GIONFH samples. GEO2R allows users to compare two or more datasets in a GEO series in order to identify DEGs under certain conditions. Probe sets without corresponding gene symbols or genes with more than one probe set were removed, respectively. A log FC (fold change) > 1 or log FC <  − 1 with an adjusted *P* value < 0.01 was considered statistically significant.

### PPI network and module analysis

STRING is a database for predicting known protein interactions. Its interactions include direct (physical) associations and indirect (functional) associations. Analysis of function and interactions between proteins with STRING may provide insights into the mechanisms involved in disease development or progression [30]. In this study, the PPI network of DEGs was constructed using the STRING database, and interactions with a composite score (the support of the data) > 0.4 were considered statistically significant. Cytoscape (3.8.0 version) is an open-source software platform for visualizing complex networks and integrating them with any type of attribute data, including bioinformatics and social network analysis [31]. Cytoscape's plugin MCODE (2.0.0 version), is used to cluster a given network based on topology to find densely connected regions (highly interconnected regions) [32]. The PPI network as well as the biological process map were drawn with cytoscape, and the most important modules in the PPI network were identified with MCODE. The selection criteria were as follows: MCODE score > 5, degree cutoff = 2, node score cutoff = 0.2, maximum depth = 100, and k score = 2. Biological process analysis of central genes and protein network interaction mapping were performed by BiNGO, a plugin of cytoscape. The relationships among the nine hub genes were explored by STRING.

### GO and KEGG enrichment analysis of DEGs

DAVIDv6.8 (http: //david.ncifcrf.gov/) [33], a visual integrated discovery database with annotation contains a complete update of knowledge base. KEGG is a database used for understanding advanced functions and practical experimental techniques in biological systems (e.g., cells, organisms and ecosystems) from molecular-level information (especially large-scale molecular datasets generated by genome sequencing and other high-throughput processes) [34]. The Gene Ontology (GO) knowledge base has the maximum information about gene function around the world, which can be recognized both by human and machine. It is also the basis for computational analysis of large-scale molecular biology and genetic experiments in biomedical research [35]. Functional enrichment analysis of DEGs was performed using the DAVID online database, and *P* < 0.05 was considered statistically significant.

### Selection and analysis of key genes

UniProt (universal protein) is a database (https://www.uniprot.org/) collecting protein resources and interconnected with other databases. It has one of the most extensive protein sequences with most comprehensive annotations so far. The 18 genes we screened were consulted using the UniProt database and annotated in lists. Functional enrichment analysis of potential genes was then performed by DAVID [28] based on up-regulated genes (EPB41, BNIP3L, KLF1, SLC7A5, C10orf10, ASXL1) and down-regulated genes (CD86, FCGR3A, FCGR2B, CYBB, TLR2, TLR4, ITGAX, TLR8, TLR1, FCGR2A, TYROBP, MS4A6A). Such analysis was also performed for individual genes (BNIP3L, ASXL1, FCGR2A, TYROBP) to search for possible protein pathways.

### Animal experiments

Thirty-six adult New Zealand white rabbits weighing 2.5–3.0 kg were purchased from the Animal Experimental Center of Jiangxi University of Traditional Chinese Medicine with license number of SYXK[gan]2004–0001. The 18 male and 18 female rabbits were single-housed with constant temperature at 20–23℃ under the humidity of 60%, and the light–dark cycle was 12 h each. After one week of adaptive feeding, those rabbits were randomly divided into three groups: blank control group (*n* = 12), model group (*n* = 12) and drug group (*n* = 12), half male and half female in each groups. Rabbits in the model group and drug group were intravenously injected with lipopolysaccharide (LPS, Sigma, USA) (10 μg/kg) via the marginal ear vein, and the second dose was administered 24 h later. After two injections of LPS, methylprednisolone sodium succinate (MSS, Huazhong Pharmaceutical, China) was injected intramuscularly at a dose of 20 mg/kg with a 24 h interval between each injection for a total of three injections. During hormone injection, 80,000 U of penicillin was intramuscularly injected into the right buttock to prevent infection (3 days) [36, 37]. After the completion of hormone injection, the drug group was intragastrical administered with simvastatin (Jingxin Pharmaceutical, China) water solution every day at a dose of 2 mg/10 ml per time for 4 weeks, the control and model groups were intragastrical administered with the same amount of saline. Magnetic resonance images of the hip joints and femoral head specimens were collected from three groups of rabbits at different time (after 2, 4, and 8 weeks). This study was conducted according to the recommendations of the National Institutes of Health Guide for the Care and Use of Laboratory Animals. The protocol was approved by the Experimental Animal Ethics Committee of the Fourth Affiliated Hospital of Nanchang University.

### Magnetic resonance imaging scanning

Magnetic resonance imaging (MRI) examination was performed at two, four, and eight weeks after the first hormone injection. 3% pentobarbital sodium solution (35 mg/kg) was injected intravenously into the ear margin of rabbits. After the anesthesia took effect, the bilateral femoral heads of all rabbits were scanned by a 3.0 T magnetic resonance imaging (MRI) scanner (Achieva TX, Philips). The parameters were as follows: T1WI (TR 700 ms, TE 45 ms), T2WI (TR 2600 ms, TE 200 ms), Stir (TR 1500 ms, TE 60 ms), slice thickness 1.2 mm, flip angle 90° and matrix (reconstruction 256 × matrix scan 256 mm). Then the MRI results of GIONFH in each group were compared.

### Extraction and anatomy of specimens

The rabbits were sacrificed by air embolism at two, four, and eight weeks after hormone injection, and the femoral heads were harvested. Washed with ice phosphate buffer saline (PBS), the left femoral heads of all rabbits were preserved in liquid nitrogen for quantitative real-time PCR. Another femoral head was collected and immediately fixed with 4% paraformaldehyde at 4 °C for 24 h for later testing.

### Pathological examination

After the femoral heads were fixed with 4% paraformaldehyde for 24 h, they were washed with 0.2 M PBS (pH 7.4) again and then decalcified in 10% ethylene diamine tetraacetic acid (EDTA) and neutralized with sodium sulphate buffer for approximately 30 days. After decalcification, the tissues were embedded in paraffin, cut into thick sections of 5 mm, and stained with hematoxylin and eosin. The osteonecrosis of the femoral head in H&E staining specimens was observed under a microscope, and the microphotos were taken. Leica Co. W550CW signal acquisition and analysis system (Weztlar, Germany) was used for observation, analysis and evaluation.

### Real-time PCR

Total RNA was extracted using rnaiso plus (Takara bio, Japan) according to the manufacturer's instructions. The RNA concentration was evaluated by A260/A280 measurement, then reverse transcription was performed, and the reverse transcription product was amplified by qPCR Expression of BNIP3L, ASXL1, FCGR2A and TYROBP were analyzed using SYBR premix Ex TaqTM II (Takara bio, Japan) on ABI 7500 rapid real-time PCR system (applied biological systems, USA). Obtain the cycle threshold (CT) value and use 2-ΔΔ the relative expression of each target gene was calculated by CT method, and the data were normalized to GAPDH expression. The primer sequence is shown in Table 1.

**Table 1:** Primer sequence information

Gene	Primer	Sequence (5′–3′)
BNIP3L	Forward	ATGTGGAAATGCACACGAGC
	Reverse	GGAAGTGGAACTCCTTGGGT
ASXL1	Forward	GAGGCTAAGACTGACTCCGC
	Reverse	CCACAGTCTCCTGAGGCAAG
FCGR2A	Forward	CAATTCTGCTGCTGTTGGCTT
	Reverse	GTCAGGGTCACCGAGTCGTC
TYROBP	Forward	ACGGTGGTGTCCAGTCGTAT
	Reverse	GATGAGCAGGGTCAACACCAG

### Statistical analysis

Quantitative data are presented as mean ± standard deviation (SD). Hardy–Weinberg genetic balance method to test population representativeness, the phenotypic differences between groups were analyzed by paired t-test to estimate the relationship between each genotype and the risk of GIONFH the odds ratio (OR), 95% confidence interval (CI) and *P* value were calculated. Data were analyzed using SPSS 23.0 software (SPSS, USA). A level of *P* < 0.05 was considered to be significant.

## Results

### Analysis of gene arrays

**Fig. 1:**
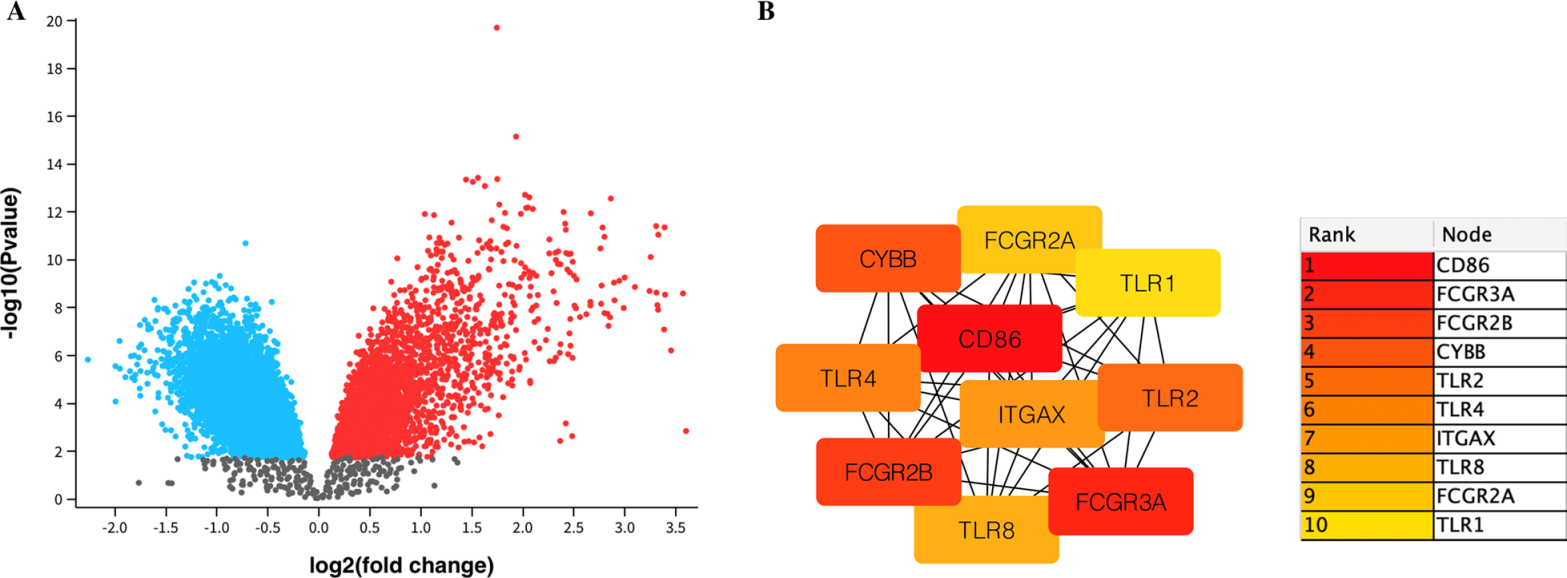
**A** Volcano diagram, the relationship between fold change and p value, blue represents down-regulation, red represents up-regulation. **B** Among the 679 differential genes, the top ten genes with the highest weight

**Fig. 2:**
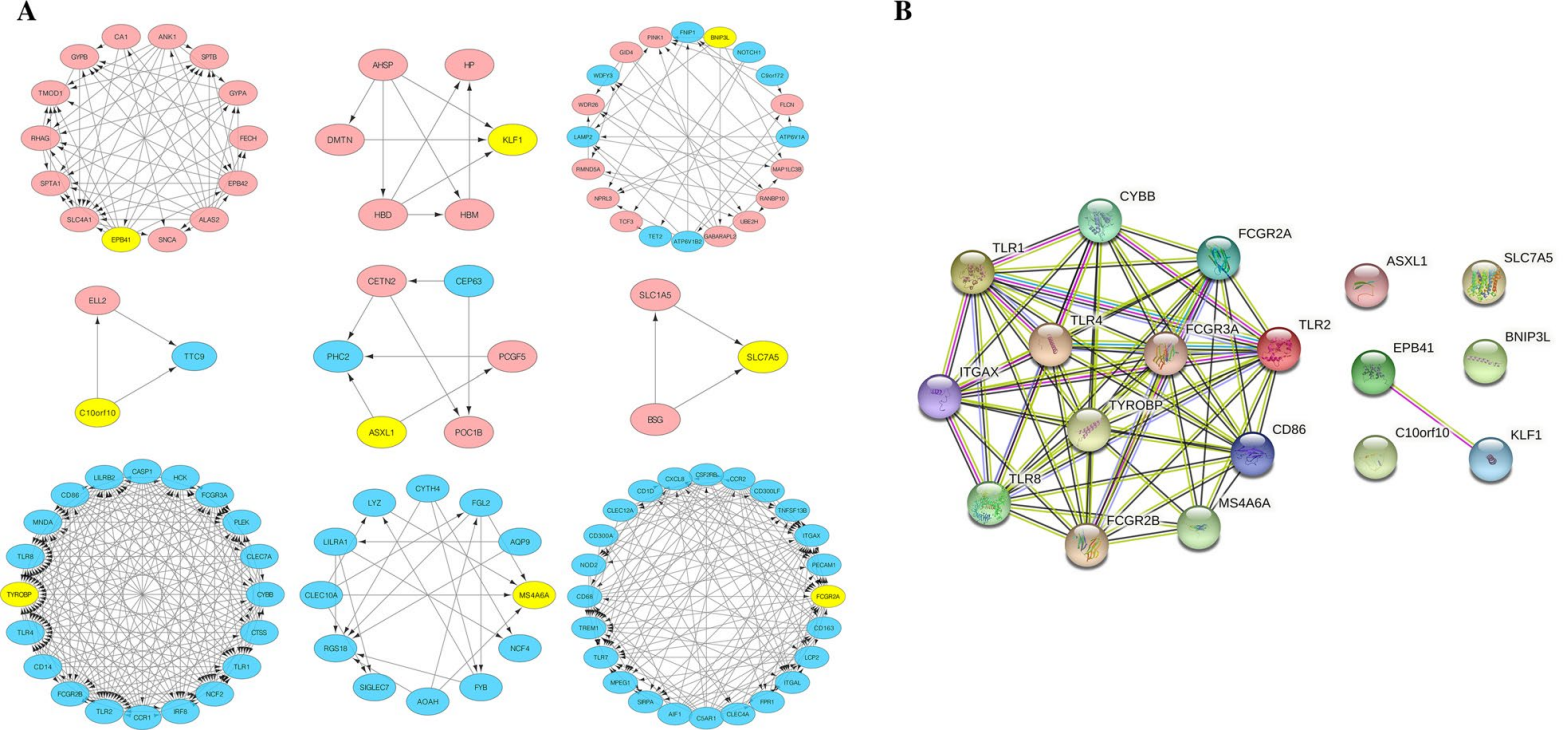
**A** There were 9 important network modules (down-regulated genes were marked in blue and up-regulated genes were marked in red), and each module had a core gene (yellow). **B** The co-expression gene network (18 gene) also showed the correlation between the 9 core gene

Through GEO2R analysis, 679 DEGs were selected including 276 up-regulated genes and 403 down-regulated genes; the volcano plot (Fig. 1A) shows the relationship between fold change and *P* value in this set of microarray groups. The 10 genes with the highest weights were all down-regulated genes by Cytoscape analysis, which were CD86, FCGR3A, FCGR2B, CYBB, TLR2, TLR4, ITGAX, TLR8, FCGR2A, and TLR1 (Fig. 1B); 9 groups were found by MCODE, and there was a hub gene in each group, of which the up-regulated genes were ASXL1, BNIP3L, C10orf10, EPB41, KLF1, and SLC7A5, and the down-regulated genes were FCGR2A, MS4A6A, and TYROBP (Fig. 2A).

### Functional enrichment analysis

**Fig. 3:**
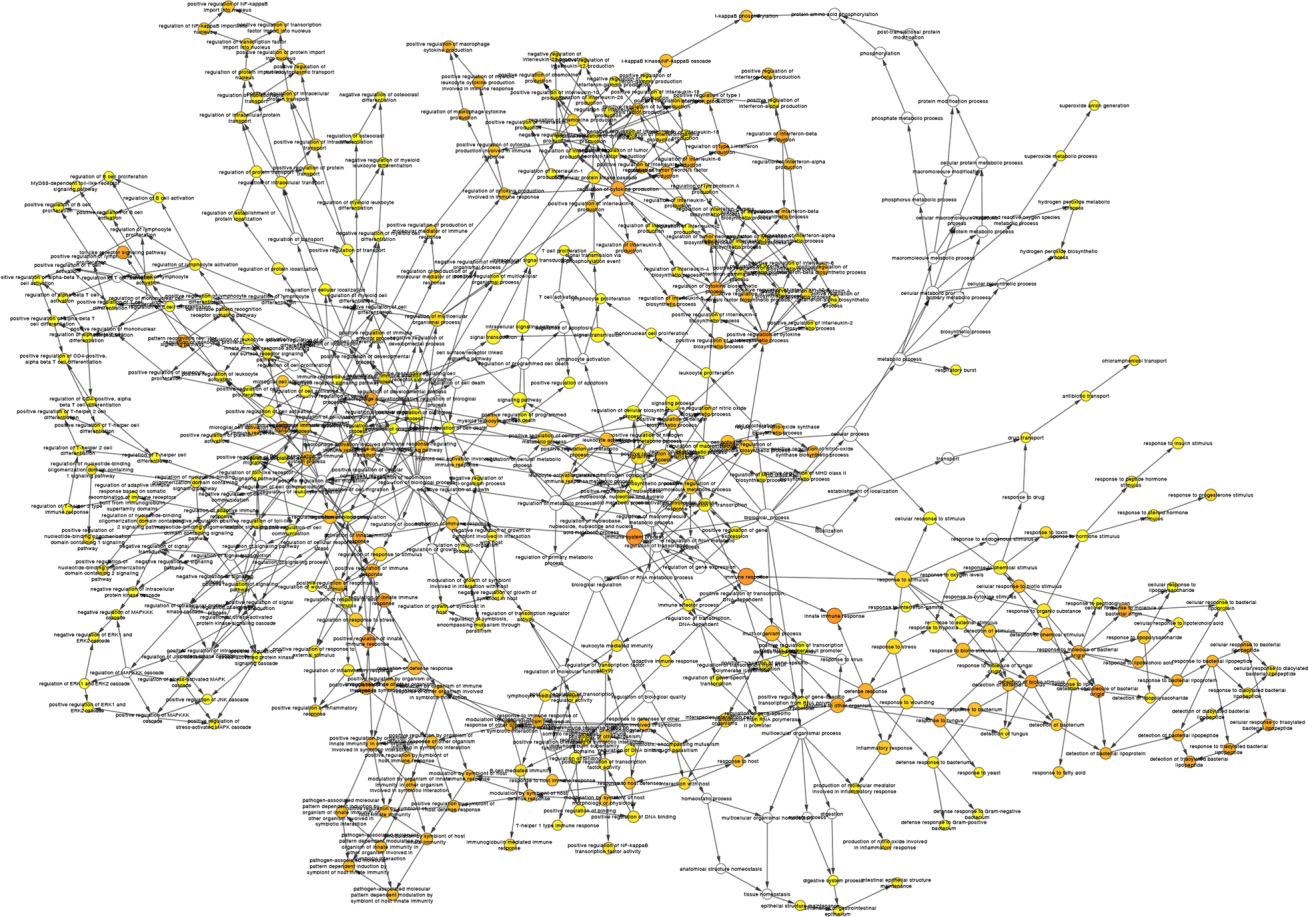
Network diagram of biological processes related to 18 genes

**Fig. 4:**
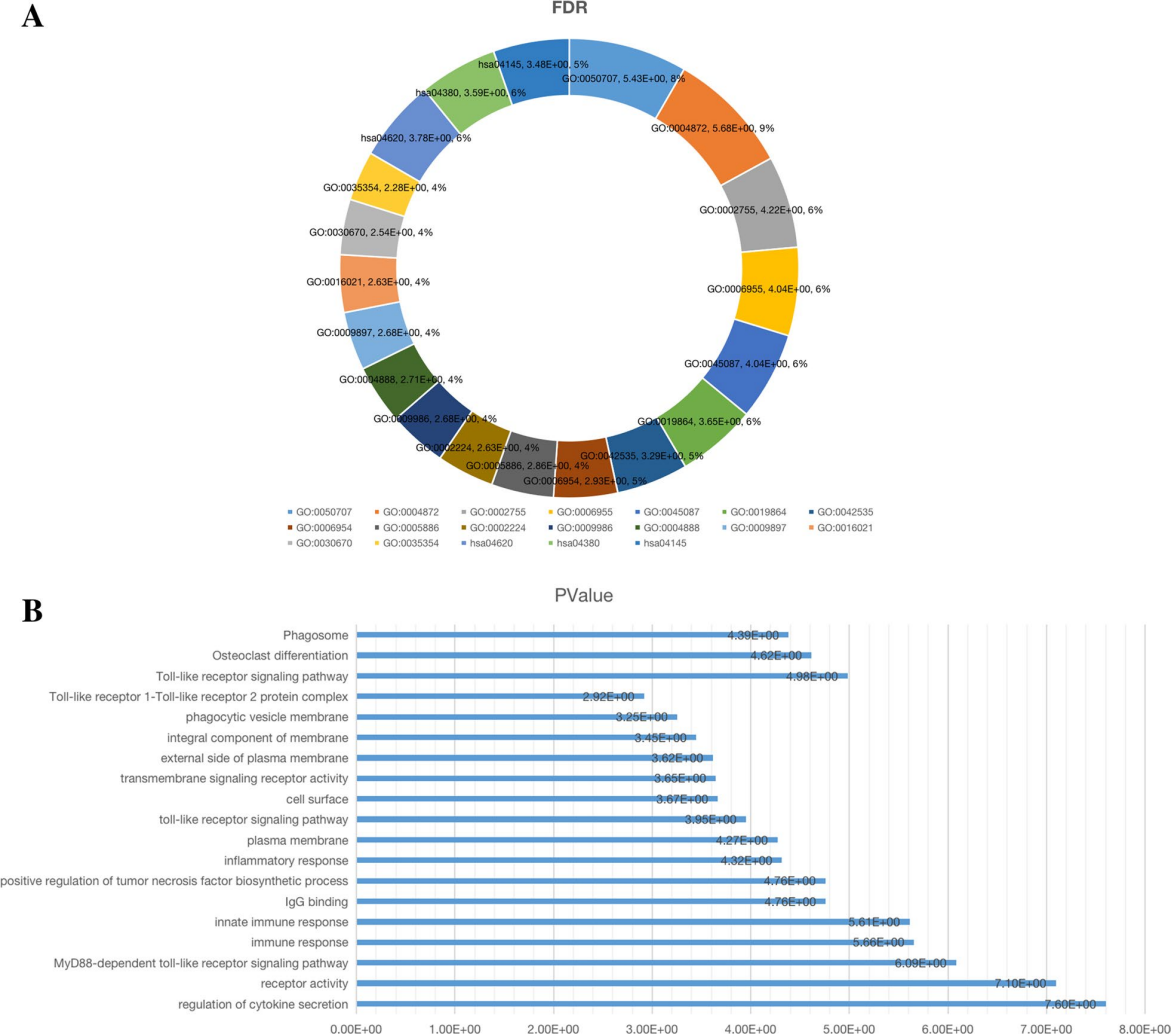
According to Table 3, we selected some important biological processes, cell structure, molecular functions and signal pathways from the functional enrichment analysis. **A** Pie chart is the relationship between items and FDR. **B** The relationship between bar graph entries and *P-*values

It should be noted that the above two groups of genes have an overlapping gene (FCGR2A), so a total of 18 differential genes are synthesized from the two groups. When exploring the relationship between the nine hub genes, some genes were found to be interrelated (Fig. 2B). A protein interaction network was drawn for the 18 differential genes to provide protein expression or potential pathways and targets that may be associated with GIONFH (Fig. 3). The results of functional enrichment analysis of 18 genes mainly focus on down-regulated genes (Table 1), of which important parts have been marked (Fig. 4). The results showed that eighteen differential genes were annotated, among which BNIP3L, ASXL1, FCGR2A and TYROBP were highly correlated with osteonecrosis (Tables 2 and 3).

**Table 2:** Enrichment Analysis (679DEG’s)

Term	Description	Count in gene set	*P*-value	FDR
*Upregulated*				
None				
*Downregulated*				
GO:0050707	Regulation of cytokine secretion	4	2.50E-08	3.70E-06
GO:0004872	Receptor activity	6	8.02E-08	2.08E-06
GO:0002755	MyD88-dependent toll-like receptor Signaling pathway	4	8.22E-07	6.08E-05
GO:0006955	Immune response	6	2.20E-06	9.02E-05
GO:0045087	Innate immune response	6	2.44E-06	9.02E-05
GO:0019864	IgG binding	3	1.73E-05	2.25E-04
GO:0042535	Positive regulation of tumor necrosis Factor biosynthetic process	3	1.75E-05	5.18E-04
GO:0006954	Inflammatory response	5	4.82E-05	0.001188048
GO:0005886	Plasma membrane	10	5.37E-05	0.001395196
GO:0002224	Toll-like receptor signaling pathway	3	1.11E-04	0.002350091
GO:0009986	Cell surface	5	2.16E-04	0.002100725
GO:0004888	Transmembrane signaling receptor activity	4	2.26E-04	0.001956399
GO:0009897	External side of plasma membrane	4	2.42E-04	0.002100725
GO:0016021	Integral component of membrane	10	3.58E-04	0.002326192
GO:0030670	Phagocytic vesicle membrane	3	5.56E-04	0.002892212
GO:0042495	Detection of triacyl bacterial lipopeptide	2	0.001190724	0.022028397
GO:0035354	Toll-like receptor 1-Toll-like receptor 2 protein complex	2	0.001206868	0.005229762
GO:0038123	Toll-like receptor TLR1:TLR2 signaling pathway	2	0.001785608	0.026426992
GO:0071727	Cellular response to triacyl bacterial lipopeptide	2	0.001785608	0.026426992
GO:0001875	Lipopolysaccharide receptor activity	2	0.002958753	0.019231894
GO:0051607	Defense response to virus	3	0.004100747	0.050269726
GO:0045359	Positive regulation of interferon-beta biosynthetic process	2	0.004161954	0.050269726
GO:0071723	Lipopeptide binding	2	0.004730221	0.024597147
GO:0050776	Regulation of immune response	3	0.004754813	0.050269726
GO:0045416	Positive regulation of interleukin-8 biosynthetic process	2	0.004755244	0.050269726
GO:0071223	Cellular response to lipoteichoic acid	2	0.005348217	0.052769071
GO:0042116	Macrophage activation	2	0.005940871	0.054953057
GO:0001774	Microglial cell activation	2	0.007125226	0.058585194
GO:0007252	I-KappaB phosphorylation	2	0.007125226	0.058585194
GO:0005887	Integral component of plasma membrane	5	0.007664838	0.028469399
GO:0051770	Positive regulation of nitric-oxide synthase biosynthetic process	2	0.007716928	0.060110805
GO:0032722	Positive regulation of chemokine production	2	0.010080561	0.074596148
GO:0042346	Positive regulation of NF-KappaB import into nucleus	2	0.012439125	0.087666214
GO:0001530	Lipopolysaccharide binding	2	0.012959674	0.056158585
GO:0032733	Positive regulation of interleukin-10 production	2	0.01361651	0.091601974
GO:0032735	Positive regulation of interleukin-12 production	2	0.014792631	0.094527539
GO:0032757	Positive regulation of interleukin-8 production	2	0.015380218	0.094527539
GO:0032728	Positive regulation of interferon-beta production	2	0.01596749	0.094527539
GO:0031226	Intrinsic component of plasma membrane	2	0.01677612	0.05452239
GO:0031663	Lipopolysaccharide-mediated signaling pathway	2	0.018899122	0.107579619
GO:0032755	Positive regulation of interleukin-6 production	2	0.026484616	0.141094616
GO:0007165	Signal transduction	4	0.027394902	0.141094616
GO:0032760	Positive regulation of tumor necrosis factor production	2	0.027646918	0.141094616
GO:0046982	Protein heterodimerization activity	3	0.029431373	0.109316528
GO:0007249	I-KappaB kinase/NF-KappaB signaling	2	0.03517149	0.173512682
GO:0071260	Cellular response to mechanical stimulus	2	0.041497457	0.197226716
GO:0050729	Positive regulation of inflammatory response	2	0.042643614	0.197226716
*Pathway*				
hsa05152	Tuberculosis	7	5.14E-08	1.65E-06
hsa04620	Toll-like receptor signaling pathway	5	1.04E-05	1.67E-04
hsa04380	Osteoclast differentiation	5	2.41E-05	2.58E-04
hsa04145	Phagosome	5	4.12E-05	3.30E-04
hsa05140	Leishmaniasis	4	1.20E-04	7.68E-04
hsa05150	Staphylococcus aureus infection	3	0.00261438	0.01394336
hsa05323	Rheumatoid arthritis	3	0.006811405	0.031137854
hsa05162	Measles	3	0.015082804	0.054398017
hsa05322	Systemic lupus erythematosus	3	0.015299442	0.054398017

**Table 3:** Annotation (18DEG’s)

Gene	Name	Notes and main functions
EPB41	Erythrocyte membrane protein band 4.1	Protein 4.1 is a major structural element of the erythrocyte membrane skeleton. It plays a key role in regulating membrane physical properties of mechanical stability and deformability by stabilizing spectrin-actin interaction
BNIP3L	Adenovirus E1B 19 kDa protein-interacting protein 3-like	Induces apoptosis. Interacts with viral and cellular anti-apoptosis proteins. Can overcome the suppressors BCL-2 and BCL-XL, although high levels of BCL-XL expression will inhibit apoptosis. Inhibits apoptosis induced by BNIP3
KLF1	Krueppel-like factor 1	Transcription regulator of erythrocyte development that probably serves as a general switch factor during erythropoiesis
SLC7A5	Large neutral amino acids transporter small subunit 1	The heterodimer with SLC3A2 functions as sodium-independent, high-affinity transporter that mediates uptake of large neutral amino acids, such as phenylalanine, tyrosine, L-DOPA, leucine, histidine, methionine and tryptophan
C10orf10	Protein DEPP1(Decidual protein induced by progesterone)	Acts as a critical modulator of FOXO3-induced autophagy via increased cellular ROS
ASXL1	Polycomb group protein ASXL1	Probable Polycomb group (PcG) protein involved in transcriptional regulation mediated by ligand-bound nuclear hormone receptors, such as retinoic acid receptors (RARs) and peroxisome proliferator-activated receptor gamma (PPARG). Acts as corepressor for PPARG and suppresses its adipocyte differentiation-inducing activity
CD86	T-lymphocyte activation antigen CD86	Receptor involved in the costimulatory signal essential for T-lymphocyte proliferation and interleukin-2 production, by binding CD28 or CTLA-4. Also involved in the regulation of B cells function, plays a role in regulating the level of IgG1 produced
FCGR3A	Low affinity immunoglobulin gamma Fc region receptor III-A	Receptor for the invariable Fc fragment of immunoglobulin gamma (IgG). Optimally activated upon binding of clustered antigen-IgG complexes displayed on cell surfaces, triggers lysis of antibody-coated cells, a process known as antibody-dependent cellular cytotoxicity (ADCC)
FCGR2B	Low affinity immunoglobulin gamma Fc region receptor II-b	Receptor for the Fc region of complexed or aggregated immunoglobulins gamma. Low affinity receptor. Involved in a variety of effector and regulatory functions, such as phagocytosis of immune complexes and modulation of antibody production by B cells
CYBB	Cytochrome b-245 heavy chain	Critical component of the membrane-bound oxidase of phagocytes that generates superoxide. It is the terminal component of a respiratory chain that transfers single electrons from cytoplasmic NADPH across the plasma membrane to molecular oxygen on the exterior
TLR2	Toll-like receptor 2	Cooperates with LY96 to mediate the innate immune response to bacterial lipoproteins and other microbial cell wall components. Acts via MYD88 and TRAF6, leading to NF-kappa-B activation, cytokine secretion and the inflammatory response. May also activate immune cells and promote apoptosis in response to the lipid moiety of lipoproteins
TLR4	Toll-like receptor 4	Cooperates with LY96 and CD14 to mediate the innate immune response to bacterial lipopolysaccharide (LPS). Acts via MYD88, TIRAP and TRAF6, leading to NF-kappa-B activation, cytokine secretion and the inflammatory response
ITGAX	Integrin alpha-X	Integrin alpha-X/beta-2 is a receptor for fibrinogen. It recognizes the sequence G-P-R in fibrinogen. It mediates cell–cell interaction during inflammatory responses. It is especially important in monocyte adhesion and chemotaxis
TLR8	Toll-like receptor 8	Endosomal receptor that plays a key role in innate and adaptive immunity. Controls host immune response against pathogens through recognition of RNA degradation products specific to microorganisms that are initially processed by RNASET2
TLR1	Toll-like receptor 1	Participates in the innate immune response to microbial agents. Specifically recognizes diacylated and triacylated lipopeptides
FCGR2A	Low affinity immunoglobulin gamma Fc region receptor II-a	Binds to the Fc region of immunoglobulins gamma. Low affinity receptor. By binding to IgG it initiates cellular responses against pathogens and soluble antigens. Promotes phagocytosis of opsonized antigens
TYROBP	TYRO protein tyrosine kinase-binding protein	Adapter protein which non-covalently associates with activating receptors found on the surface of a variety of immune cells to mediate signaling and cell activation following ligand binding by the receptors. TYROBP is tyrosine-phosphorylated in the ITAM domain following ligand binding by the associated receptors which leads to activation of additional tyrosine kinases and subsequent cell activation. Required for the activation of myeloid cells mediated by the CLEC5A/MDL1 receptor. Associates with SIRPB1 to mediate activation of myeloid cells, such as monocytes and dendritic cells
MS4A6A	Membrane-spanning 4-domains subfamily A member 6A	May be involved in signal transduction as a component of a multimeric receptor complex

### Magnetic resonance imaging results

**Fig. 5:**
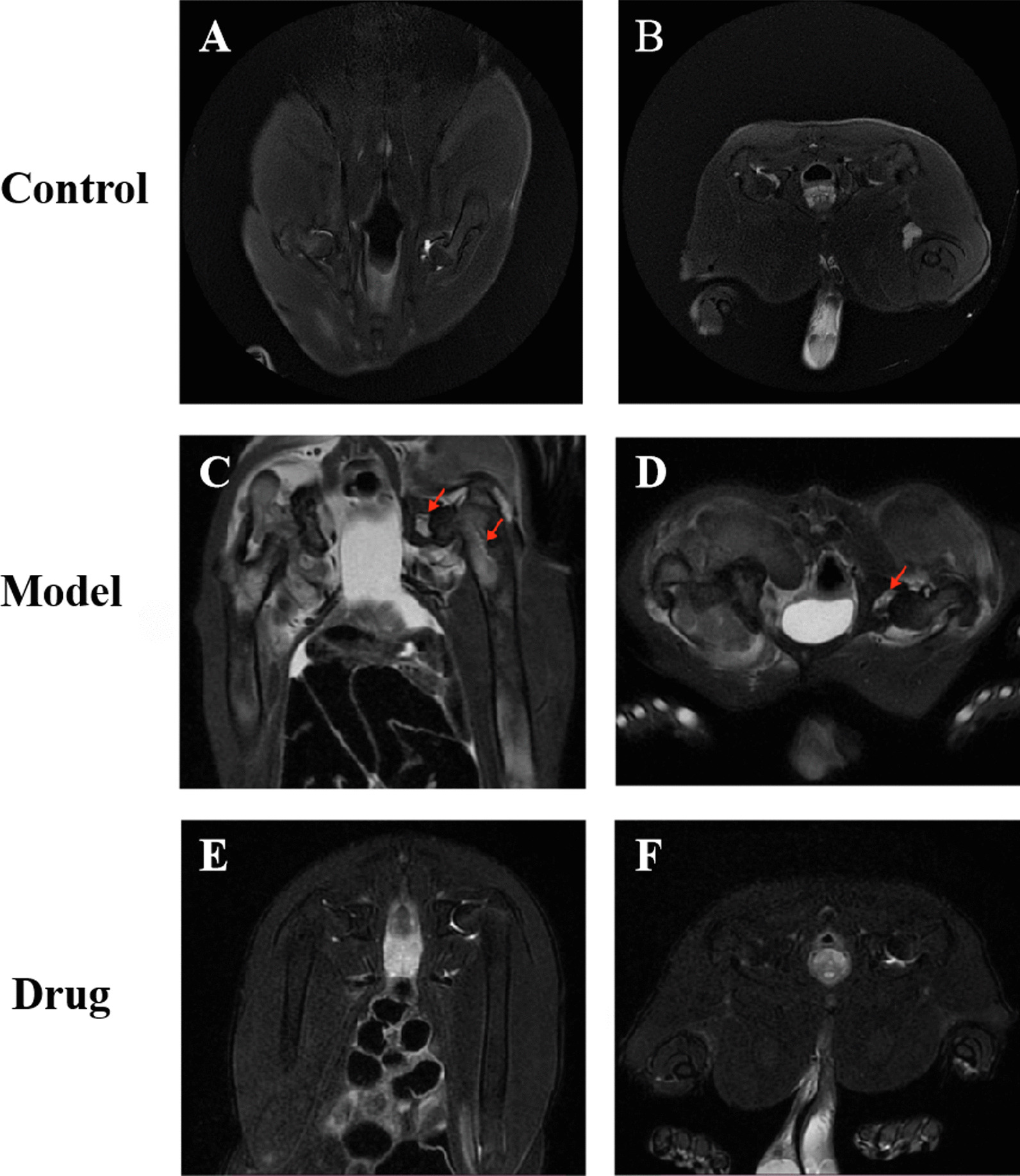
Analysis of bone structure of the rabbit femoral head by hip magnetic resonance coronal and traverse scanning. The representative images of rabbit bilateral femoral heads MRI scanning in the control group (**A**, **B**), model group (**C**, **D**) and drug group (**E**, **F**).The red arrow indicated slight collapse and flattening of femoral heads, edema signals in joint cavities and bone marrow

Magnetic resonance imaging (MRI) is considered the most sensitive, specific modality and golden standard for the diagnosis and evaluation of GIONFH. It was used to analyze the imaging changes in the rabbits’ femoral heads. The results of T2 weighted MRI showed that in the blank control group, the shape of bilateral femoral heads was regular, the surface was smooth, no abnormal signals were found, and the bilateral hip joint space was normal (Fig. 5 A, B). As for the model group after 8 weeks, the T2-weighted images showed spotty, heterogeneous high signal intensity, slight collapse and flattening of bilateral femoral heads, edema signals in bilateral joint cavities (red arrow), and uneven joint spaces (Fig. 5 C, D). In the drug group, the joint space were normal, no obvious effusion was in the hip cavity, the epiphyseal line of the femoral heads were slightly blurred, and no obvious abnormal signals were found (Fig. 5 E, F). These results indicated that steroid hormone-induced osteonecrosis of the femoral head, however, simvastatin could exert a protective effect on GIONFH in vivo.

### Histopathological analysis H&E staining

**Fig. 6:**
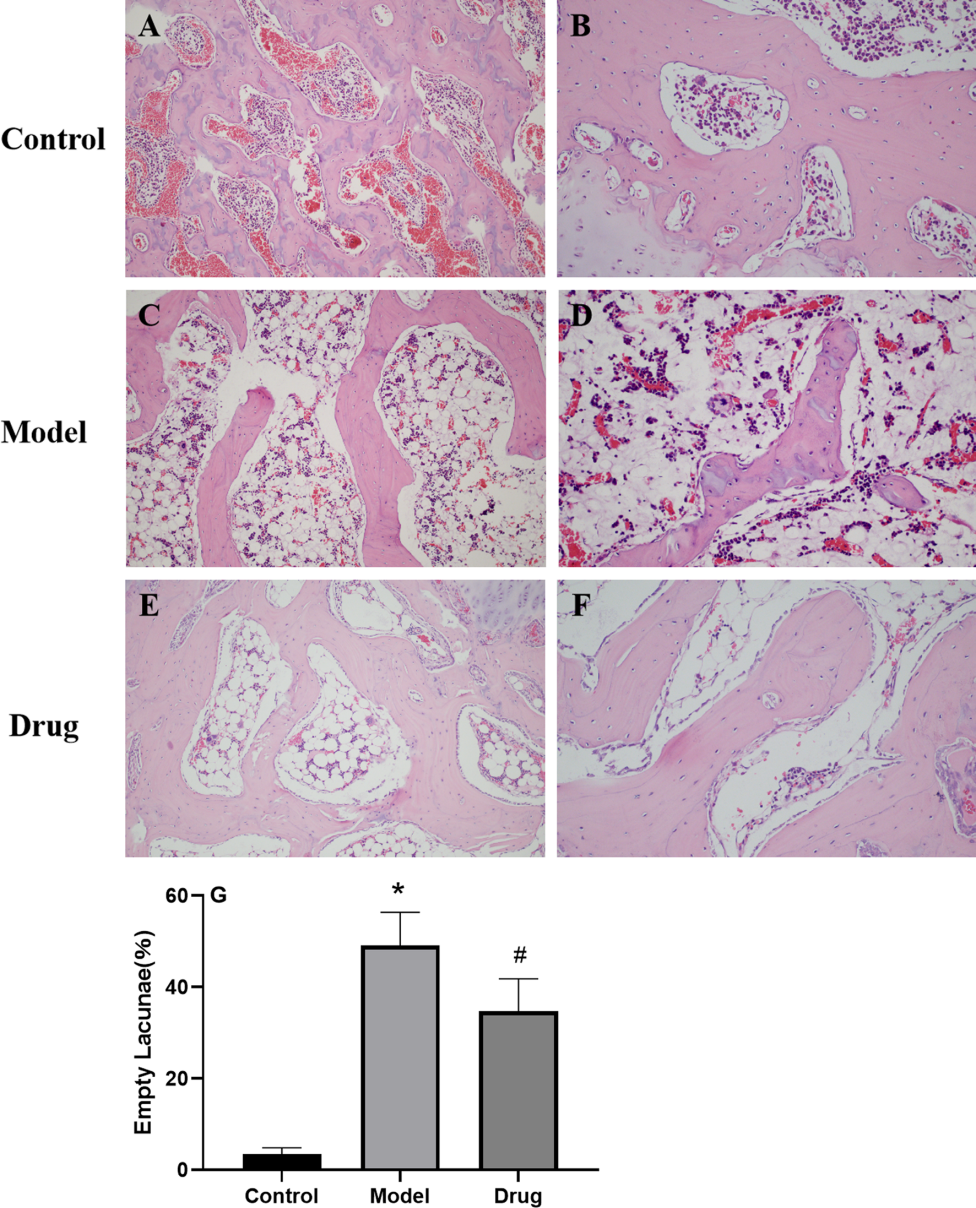
Histopathological H&E staining of GIONFH in vivo. The representative images of H&E staining from the femoral heads in three groups. There were no empty lacunae in the femoral head of the control group (**A**, **B**). A large number of empty bone lacunae and necrotic bone marrow cells are visible in the model group (**C**, **D**), while there were few empty bone lacunae in the drug group (**E**, **F**). Magnification: × 200 (**A**, **C** and **E**), × 400 (**B**, **D** and **F**). The histogram shows the proportion of empty bone lacunae in the model group, which are significantly higher than those in the control group and drug group (**G**).The data are expressed as the means ± S.D. Significant differences between different groups are indicated as* *p* < 0.05 vs. the control group and # *p* < 0.05 vs. the model group

Based on the diagnostic criteria of GIONFH, we evaluated femoral head specimens of rabbits using H&E staining. In this study, none of the rabbits in the control group developed osteonecrosis. The bone trabeculas were regularly arrayed, with complete structure, clearly visible osteocytes and a few empty lacunaes (Fig. 6 A, B). In the model group, typical osteonecrosis occurred. The lipocytes were enlarged. Bone trabeculas turned thinner, and many empty lacunaes were observed (Fig. 6 C, D). In the drug group, there were a few lipocytes, and no apparent necrotic debris was noted. The bone trabeculas regularly arrayed, and only a few empty lacunaes were observed (Fig. 6 E, F).

### Expression of the key differential genes in the rabbit model of GIONFH

**Fig. 7:**
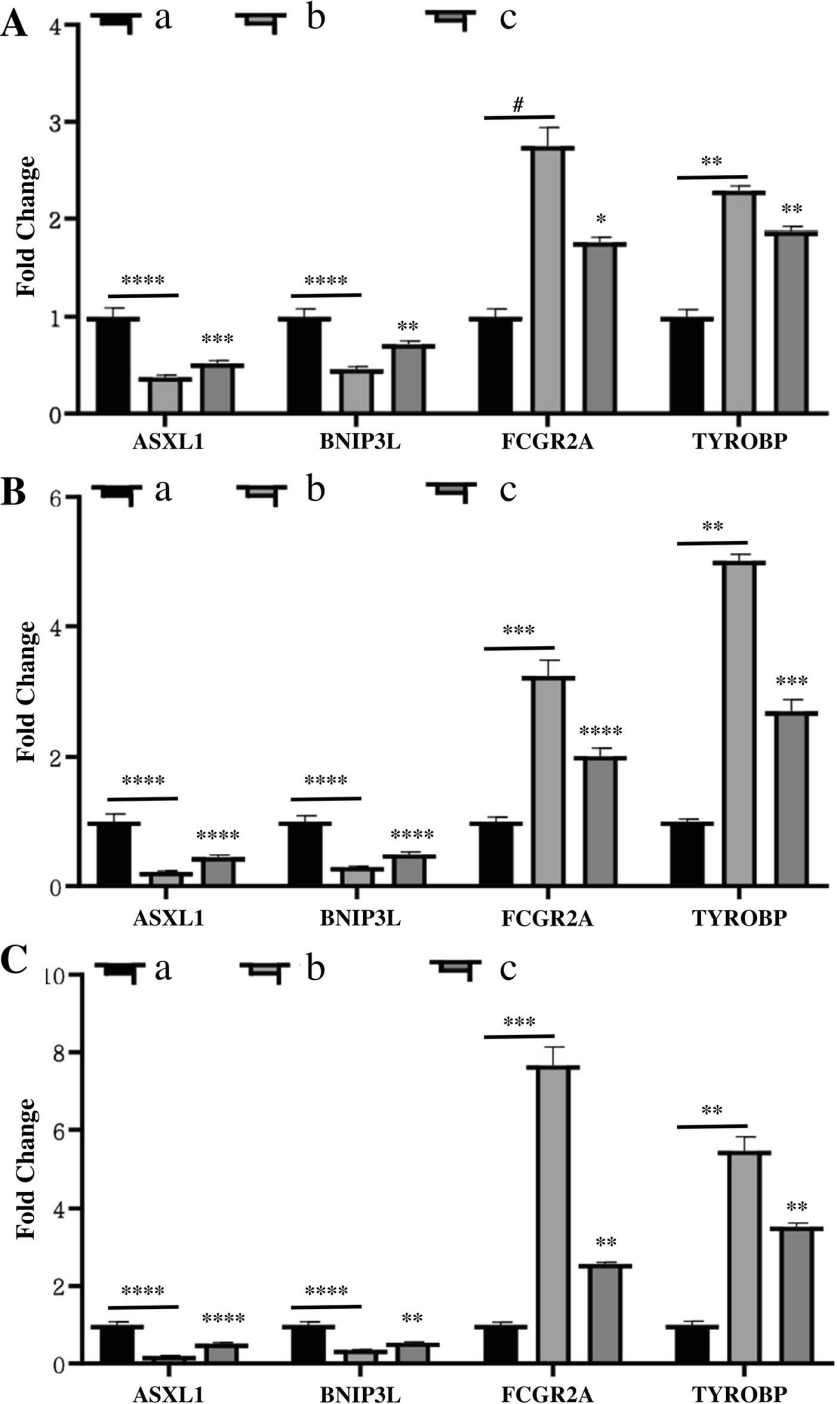
Real-time PCR assay showed the expression of ASXL1, BNIP3L, FCGR2A and TYROBP mRNA in the control group (**a**), model group (**b**) and drug group (**c**) at 2 weeks (**A**), 4 weeks (**B**), and 8 weeks (**C**). The underlined asterisk (*) represents the comparison between the model group and the control group; The asterisk (*) represents the comparison between the drug group and the control group. # means *P* > 0.05, * means 0.01 < *P* < 0.05, ** means *P* < 0.01, *** means *P* < 0.001, **** means *P* < 0.0001. (group a black histogram; group b light grey histogram; group c dark grey histogram)

In order to investigate the expression of the key differential genes related to GIONFH in rabbits, real-time PCR was performed to measure the expression level of the ASXL1, BNIP3L, FCGR2A and TYROBP at different time points (after 2, 4, and 8 weeks). After 2 weeks, the results showed that the expression level of ASXL1 and BNIP3L were significantly decreased and the TYROBP robust increased in the model group compared to the blank control group (Fig. 7 A). But this effect was abolished when the rabbits were treated with simvastatin in the drug group. Similarly, after 4 and 8 weeks, the expression level of ASXL1 and BNIP3L decreased significantly in the model group compared to the blank control group, while the FCGR2A and TYROBP increased remarkable (Fig. 7 B, C). It should be noted that this effect was blocked by simvastatin in the drug group. Together, these data suggested that the four key differential genes ASXL1, BNIP3L, FCGR2A and TYROBP were significantly associated with osteonecrosis of the femoral head.

## Discussion

Although steroid hormones play an important role in the treatment of many diseases, they produce a series of side effects. For example, long-term or extensive use of steroid hormone may cause GIONFH [38]. Previous studies have shown that without early intervention, 80% of patients will develop a collapsed femoral head and significantly impaired hip function within 1–5 years of onset [39]. Such treatments for collapsed and necrotic femoral head as core decompression, Osteotomies, bone pedicled vascularized or autologous stem cell implantation are all invasive operations [40–43]. After the operations patients are very likely to have unbalanced bone resorption, which, as is well known, can be enhanced by corticosteroids [42]. Due to the negative effect in the long run, total hip arthroplasty (THA) is eventually an inevitable option. Therefore, more and more researchers are devoted to the study of early diagnosis and preventive drugs for GIONFH.

Bioinformatics analysis and microarray technology at the genome level are mostly adopted in tumor research. At first, the researchers sequenced the tumor sample cells of human, and uploaded the sequenced gene array to the gene bank. Then, a variety of sequencing results were downloaded from the gene bank by other researchers and tens of thousands of gene fragments were obtained after researchers’ classification and annotation. At last, key genes or mutated genes were identified from these gene fragments for research to discover the disease pathogenic mechanism and therapeutic targets [44, 45]. With the help of the analysis platform of lipid metabolomics technology, our research group has studied the variation trend of the metabolic profile spectrum of animal samples of GIONFH before and after the intervention, and confirmed that GIONFH is closely related to the expression of lipid metabolism gene. Moreover, animal experimental studies have confirmed that the application of lipid-lowering drugs could effectively improve the occurrence and disease progression of GIONFH [20, 21]. In this study, we obtained 679 differentially expressed genes by screening between the gene array of human GIONFH and non-GIONFH. In the screening analysis of 679 differentially expressed genes, the following two algorithm were used to identify the potential key genes. Ten weight genes were obtained by MCC (maximum clique centrality) algorithm. According to the statistics on its official website, the key networks captured by MCC algorithm will bring new insights into the basic regulatory networks and protein drug targets for experimental biologists. In addition, we also used the MCODE (molecular complex detection) plug-in to obtain nine gene groups. Despite the complexity of running the code of MCODE, this method has been used by many medical researchers for many times, which is enough to verify its reliability. Therefore, we used this method to obtain 18 potential genes to be verified.

We compared and analyzed the correlation between the related proteins expressed by the 18 genes and steroid-induced femoral head necrosis one by one. TYROBP and FCGR2A were associated with osteonecrosis at different sites in the same pathway, among which the mutation of TYROBP-DAP12 encodes membrane receptor component cells in natural killer cells and myeloid cells, which has been confirmed by Juha Paloneva et al. [46], meaning TYROBP-mediated signaling pathway played an vital role in human bone tissue. Another study by Juha Paloneva and her team [47] also demonstrated an important role for the DAP12-TREM2 signaling complex in the differentiation and function of osteoclasts. This undoubtedly highlights the importance of the high expression of TYROBP in the rabbit GIONFH model tissue. FCGR2A acts as an Fc region-binding receptor of immunoglobulin γ and has been found to be associated with many inflammatory diseases [48]. FcgammaRIIa (FcγRII) is a multivalent IgG receptor expressed primarily by myeloid cells, and its binding to lipid rafts (microdomains rich in cholesterol and sphingolipids) is critical for efficient signaling of the pathway [49]. Yang et al. [50] found in the study of myeloma that hepatocytes increase the secretion of CRP (C-reactive protein) in response to myeloma-derived cytokines, and the binding of CRP to FCGR2 on the surface of myeloma cells activates myeloma cells to promote osteoclastogenesis and bone destruction in vivo. It has also been shown that aggregation of FcgammaR on human bone marrow cells leads to tyrosine phosphorylation of inositol polyphosphate containing SHIP-2 (SRC homology 2 domain), which reveals a new regulatory role of the expression and function of inositol phosphatase SHIP-2 on FcgammaR mediated activation of human bone marrow cells [51]. These studies suggested that the FCGR2A we screened out might be highly associated with GIONFH and thus needs further study and validation. By consulting the literature, we could not find that the BNIP3L and ASXL1 were directly related to the occurrence of osteonecrosis. However, the protein encoded by BNIP3L/NIX (NIX named Bcl-2/E1B19 kDa-interacting protein 3-like, which is based on 56% homology with BNIP3) is itself a pro-apoptotic protein. BNIP3L/NIX can not be detected in normal tissues, and they were found in a variety of organelle cultures in hypoxia-induced experiments [52, 53]. The Bcl-2 gene family of hypoxia-induced NIX is expressed during erythropoiesis, and the researchers found that NIX is highly regulated during erythroid end-stage maturation [54]. This shows that NIX associated with BNIP3L can cause erythropenia under hypoxic conditions, which coincides with the ischemia and hypoxia that cause osteonecrosis of the femoral head. ASXL1 belongs to an oncogene with unstable genome and recent studies have shown that mutations in ASXL1 are found in hematopoietic cells of various myeloid tumors such as myelodysplastic syndrome and chronic myelomonocytic leukemia [55, 56]. Our bold guess here is: are patients with mutations in ASXL1 more likely to develop GIONFH? While more evidence is needed. Therefore, we selected these four core genes with the highest correlation to GIONFH as research targets.

Through the induction of hormone and endotoxin, rabbit femoral head necrosis was caused. In the experiment later, we fully proved the success of animal model establishment through the diagnostic criteria of magnetic resonance and pathological examination. In the experimental group, we took four New Zealand white rabbits at different time points of the second, fourth and eighth weeks after hormone use for MRI examination, and dissect their femoral head specimens for pathological examination and gene detection. Through the experiment, we were pleased to find that the quantitative expression of the four core genes, compared with the blank control group, in the animal samples of steroid-induced necrosis of the femoral head, the expression of ASXL1 and BNP3L is reduced, while the expression of FCGR2A and TYROBP is increased and hypolipidemic drugs can play a certain role in prevention and treatment of steroid-induced necrosis of femoral head in model animals. This result not only confirms that the core genes screened in the earlier stage do have an important relationship with the research purpose, and it is very important for early diagnosis with steroid-induced necrosis of femoral head.

The prevalence of GIONFH is increasing, and people are diagnosed with it at a much earlier age. With the loss of function of the hip joint, it has brought great harm to the physical and mental health of patients [57, 58]. We hope to find the key biomarkers of pathogenicity through bioinformatics analysis and microarray technology at genome level, so as to detect the biomarkers for early diagnosis of GIONFH or to find therapeutic targets for early prevention or effective treatment to avoid further harm.

In this study, we aimed to screen and identify DEGs that might be involved the GIONFH, 679 differential genes were screened using the public gene expression comprehensive dataset. These 18 genes might be associated with the occurrence of GIONFH. The animal model was successfully established, the expression of genes preliminarily confirmed the results of gene microarray analysis. We speculated that the expression of ASXL1, BNIP3L, FCGR2A and TYROBP were significantly associated with GIONFH, which was expected to become a new perspective for the study of the pathogenesis of GIONFH.

## Data Availability

The datasets used and/or analyzed during the current study are available from the corresponding author on reasonable request.
